# mTOR pathway occupies a central role in the emergence of latent cancer cells

**DOI:** 10.1038/s41419-024-06547-3

**Published:** 2024-02-28

**Authors:** Kseniia V. Aleksandrova, Mikhail L. Vorobev, Irina I. Suvorova

**Affiliations:** Institute of Cytology, Russian Academy of Sciences, St. Petersburg, Russian Federation

**Keywords:** Translation, Cancer stem cells, Metastasis

## Abstract

The current focus in oncology research is the translational control of cancer cells as a major mechanism of cellular plasticity. Recent evidence has prompted a reevaluation of the role of the mTOR pathway in cancer development leading to new conclusions. The mechanistic mTOR inhibition is well known to be a tool for generating quiescent stem cells and cancer cells. In response to mTOR suppression, quiescent cancer cells dynamically change their proteome, triggering alternative non-canonical translation mechanisms. The shift to selective translation may have clinical relevance, since quiescent tumor cells can acquire new phenotypical features. This review provides new insights into the patterns of mTOR functioning in quiescent cancer cells, enhancing our current understanding of the biology of latent metastasis.

## Facts

- The mTOR kinase inhibition is an obligatory molecular event for entry into a state of cellular hibernation in stem cells and cancer cells
- The mTOR pathway inhibition leads to suppression of global protein synthesis and activation of alternative translation pathways through eIF2α phosphorylation, predominantly ensuring the synthesis of mRNAs with uORFs in cells under non-dividing conditions
- uORFs are commonly found in the structure of oncogenic mRNAs, representing a potential pathway for the emergence of a stem-like phenotype in quiescent cancer cells
- Persistent cancer cells lacking the mTOR activity are quiescent cancer cells which accumulate mRNAs with uORFs, thus augmenting oncogenic potential, and can resume proliferation as cancer stem cells

## Open questions

- Does phenotypic evolution of cancer cells occur through the mTOR inhibition-based dormant transitions?
- What epigenetic mutations must occur in cancer cells to effectively block the mTOR pathway under specific stimuli?
- What causes the mTOR pathway to be re-activated in latent metastatic cancer cells?

## Introduction

Understanding the biology of dormant cancer cells contributing to all types of cancer recurrences remains challenging. Numerous studies have demonstrated that cancer cells transiently enter a latent phenotypical state that permits them to remain dormant in the adult organism for extended periods of time and thus to resist radio- and chemotherapy. BrdU incorporation and Ki67 antibody staining are universal markers of cellular proliferation clinically used to predict disease recurrence and overall prognosis. However, these are insufficient for effective residual disease diagnostics. The lack of relevant markers is mainly due to a poor understanding of the dormant cancer cell biology. First, it remains unclear to what extent cellular dormancy strategy is adaptive (i.e., phenotypical) or genetic. In a recent work, Rehman and colleagues showed that all colorectal tumor cells are competent to enter the dormant state [1]. On the other hand, various tumor cell lines, such as the squamous cell carcinoma cell line HEp3, spontaneously enter a noncycling G_0_ state under in vivo conditions after prolonged in vitro passages [2, 3]. These data indicate that some mutations in cancer cells may account for the susceptibility to the induction of a dormant phenotype under certain microenvironment conditions. Second, there is an incomplete understanding of the correlation between the cancer cell-reproduced dormancy and the normal reproductive strategy of stem cell quiescence, which forms numerous quiescent populations in the brain, skin, lungs, colon, bone marrow, and other tissues [4]. Finally, there is still no precise definition of the dormant state of cancer cells. The current definition includes low cycling, transient cell cycle arrest, prolonged cell cycle block, quiescence, deep hibernation, senescence, reversible/nonreversible radio- and chemoresistance. Should one interpret the observed types of proliferative restrictions as the same form of the dormant state, one cannot correctly identify the molecular, physiological, and phenotypical features of dormant cancer cells, existing in transiently arrested, quiescent, and senescent states. Recent investigations indicate mTOR signaling inhibition to be a central node for the transition of stem cells [5–11] and, in particular, cancer cells [1, 8, 12–16] to a quiescent state. The mechanistic inhibition of the mTOR pathway was demonstrated to be an effective strategy for generating a quiescent state in embryonic stem cells [9, 10], stem cells [8, 17], and cancer cells [1, 13, 15, 16]. Accordingly, dormant cells can be distinguished from cells temporarily arrested in cycling based on activity of mTOR protein. This review analyzes the role of the mTOR pathway in quiescent cancer cells with the purpose of elucidating the biology of dormant cancer cells.

## Two types of reversible cell dormancy: temporarily arrested cells and quiescent cells

The transcriptional regulator NR2F1 is known as a potential clinical marker of dormant cancer cells, and quiescent disseminated tumor cells (DTCs) of different cancers can be found using NR2F1 antibodies [14, 18–21]. The activation of the NR2F1 gene can occur during epigenetic chemotherapy, giving tumor cells the ability to form latent phenotype [19]. Dormant state induction in cancer cells via NR2F1 overexpression has been demonstrated in numerous experiments in vitro, in patient-derived organoids, and in vivo [14, 20]. Another transcriptional factor, such as NFATC4, has been recognized as a potential marker of dormant tumor cells in ovarian cancer [22]. NFATC4 was shown to be enriched in a population of slowly dividing cancer stem cells of ovarian cancer. Treatment of cancer cells with cisplatin resulted in NFATC4 nuclear translocation and activation of the NFATC4 pathway, initiating a program of cellular dormancy [22]. NFATC4-positive cancer cells were characterized by decreased proliferation, G_0_ cell cycle arrest, reduced cell size, and chemotherapy resistance in vitro and in vivo [22]. Recently, another transcriptional factor ZFP281 was found to lock early DTCs in a dormant state [23], increasing the number of newly identified transcription factors involved in cancer cell dormancy. Accordingly, an obvious question arises: is there any molecular cross road where the functions of these and other transcription factors involved in establishing of cellular dormancy converge? Accumulated data indicate that both NFATC4 and NR2F1 can effectively inhibit the MYC transcriptional module [19, 22]. MYC-induced transcriptomes are well known to represent central transcriptional hubs in the control of growth and proliferation of all cell types, therefore MYC silencing constitutes a tremendous molecular event in cycling cells. NR2F1 overexpression successfully inhibited the proliferation capacity of MMTV-MYC tumor cells obtained from the MMTV-MYC transgenic mouse model characterized by enforced c-MYC expression [19]. Inducible activation of NFATC4 also resulted in the effective suppression of MYC expression in tumor cells [22]. Interestingly, reactivation of MYC, following the upregulation of NFATC4, partially inhibited the quiescent phenotype and failed to fully restore proliferative phenotype in cancer cells [22]. MYC suppression is the molecular basis of cellular quiescence, found in embryonic stem cells (ESCs) [9, 24], stem cells, and cancer cells [1, 13]. Thus, the ability of transcriptional factors to effectively suppress the MYC module may define their affiliation to the cellular dormancy program.

The MYC signaling regulates ESC entry into diapause in vitro and in vivo [9, 24]. Diapause is a stage of reversible proliferation blockage of the inner cell mass of blastocysts that can be reproduced in vivo and in murine stem cells in vitro [10, 24]. Residual tumor cells remaining in patients after treatment and detected in preclinical models are characterized by significant suppression of MYC [1, 13], and depleting MYC in tumor cells leads to a dormant diapause-like adaptations allowing them to survive under chemotherapy [13]. MYC suppression was detected in long-term quiescent hematopoietic stem cells (HSCs) [25] and long-term quiescent neural stem cells (NSCs) [26, 27]. It is well known that nonproliferating stem cells can be in two distinct quiescent states, namely long-term dormancy and short-term dormancy, distinguished signaling pathways and transcriptional programs [26–28]. MYC activation was observed in a subpopulation of dormant NSCs ready for activation but not in deeply quiescent NSCs [26, 27]. Deeply dormant HSCs also lack activated MYC, with MYC expression detected in slow-cycling and transiently arrested cells [25]. The point is that deeply dormant stem cells are highly catabolic, and MYC, as anabolic driver, disrupts the catabolic regime in noncycling stem cells, counteracting the constitutive lysosomal flux governed by TFEB [25, 29–31]. The TFEB and MYC programs determine the balance between catabolic and anabolic processes required to activate and inhibit the long-term dormancy program of HSCs [25]. Accordingly, the TFEB-regulated lysosomal biogenesis is upregulated only after the MYC inhibition in HSCs during the transition to long-term hibernation [25]. TFEB is a known master regulator of degradative intracellular processes that enables phenotypic cellular plasticity via cellular clearance [32, 33]. The TFEB activation in noncycling cells may reflect the transition of cells into a new phenotypical adaptation designed for prolonged dormancy. Giving the above, Ki67-positive tumor cells retaining the MYC activity [34] cannot be interpreted as quiescent cells because theirs intracellular machinery was not reorganized according to demands for a metabolic profile of quiescence. These cells should be referred to as temporarily suspended cells that retain their original characteristics. Thus, the following conclusions can be drawn: cellular quiescence, representing a separate phenotypic state, is not the same as temporary cell cycle arrest; the MYC activity determines the transition from cell cycle block to quiescence in stem cells and cancer cells; quiescent cancer cells can acquire specific intracellular traits enabling their detection.

## Quiescent cells are characterized by the suppressed mTOR signaling

**Fig. 1:**
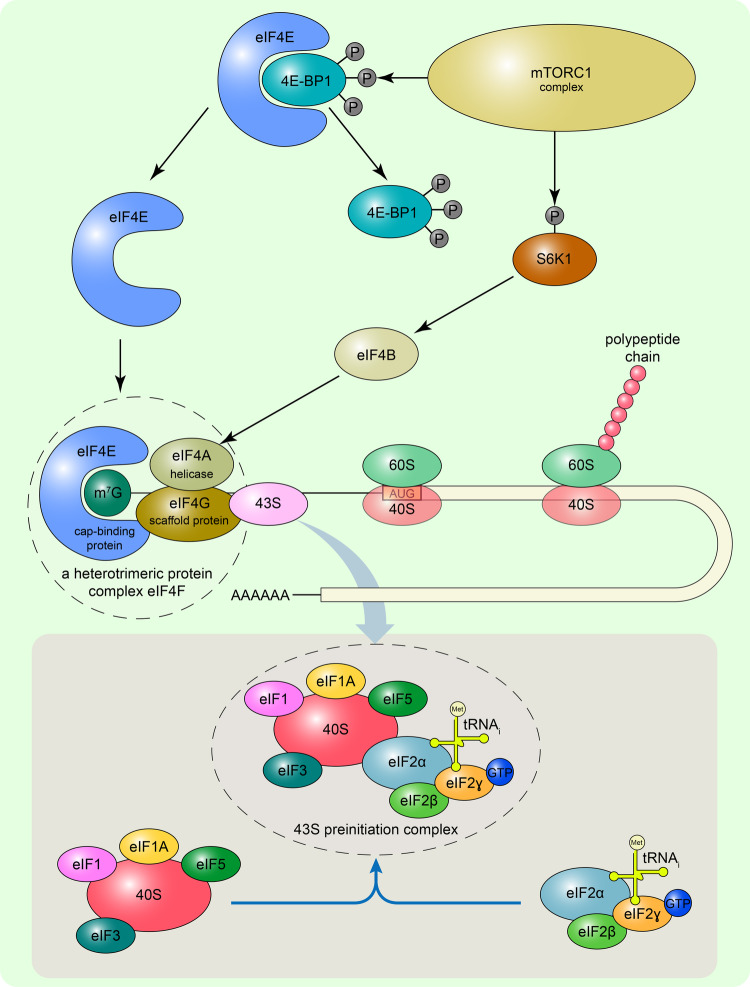
The mTOR-regulated cap-dependent translation. The mTORC1 complex phosphorylates the 4E-BP1, which then releases cap-binding protein eIF4E. Along with the scaffold protein eIF4G, and helicase eIF4A, eIF4E recognizes the 5’7-methylguanosine cap (m7G) to form the eIF4F complex. The helicase activity of eIF4A, which unwinds secondary structures, is regulated by the mTOR-dependent phosphorylation of the S6K1 protein, which dissociates from eIF4B protein, releasing it from suppression. After the eIF4F complex assembly, the 43S ribosomal preinitiation complex is attracted to the unwound mRNA area. Further, the 43 S ribosomal complex moves along the 5’UTR of mRNA and scans for the AUG start codon. After the recognition of the start codon, the 60S ribosomal subunit is attracted to form the 80S translationally competent ribosome. The 43S ribosomal preinitiation complex comprises a 40S ribosomal subunit associated with eIF1, eIF1A, eIF3, eIF5, and the eIF2-GTP-tRNA_i_^Met^ ternary complex. eIF2 is a stable heterotrimeric protein, consisting of α-, β-, and γ-subunits, that binds GTP and an initiator tRNA (tRNA_i_) carrying formyl-methionine to ribosomes (tRNA_i_^fMet^). Only the GTP-bound form of eIF2 can bind tRNA_i_^fMet^.

The activity of the serine/threonine kinase mTOR is critical for the proliferation of all cell types. Due to the unique molecular structure enabling the formation of multisubunit complexes, mTOR is intercalated into numerous cellular processes related to protein synthesis. The mTOR catalytic subunit is currently known to be part of the mTORC1 and mTORC2 complexes and is assumed to be part of mTORC3 and mTORC4 complexes [35, 36]. Owing to its specific binding with rapamycin, mTORC1 was found to play a central role in global protein synthesis. Canonical mTORC1 signaling includes the 4E-BP1 and S6K1 phosphorylation events (Fig. 1). The 4E-BP1 phosphorylation prevents the negative binding of 4E-BP1 with eIF4E, allowing the assembly of translation initiation factors at the 5’ 7-methylguanosine (m7G) cap at the mRNA terminus. The S6K1 phosphorylation leads to the S6K1 activation and transmission of upstream mTOR-dependent signals to eIF4B, which stimulates the helicase activity of eIF4A to unwind the inhibitory secondary structure in the 5’ untranslated region of mRNAs [37] (Fig. 1). S6K1 also signals to ribosomal protein S6 for providing the translation of a subgroup of transcripts, 5’ terminal oligopyrimidine (TOP) mRNAs encoding ribosomal proteins and other components of translational apparatus [38]. mTORC1-mediated cap-dependent translation is typically considered to be responsible for global protein synthesis in cells in response to nutrient and growth factors [39].

It can be assumed that trophic functions of mTOR involved in cell growth and division have to be significantly inhibited in cells under cell cycle arrest and, to an even greater extent, in quiescent cells. The dormant states of various stem cell types, including ESCs [9, 10], hematopoietic [5], neural [11], satellite [7], oocytes [6], and hair follicle stem cells [8, 17], were demonstrated to be accompanied by significant inhibition of mTOR signaling. According to recent data, dormant cancer cells are also characterized by suppressed mTOR kinase activity [1, 12–14]. Under the pharmacologic activation of the transcriptional factor NR2F1, a master regulator of tumor cell dormancy, cancer cells acquire a dormant phenotype associated with the inhibition of cell cycle progression and mTOR signaling [14]. Therefore, the mTOR pathway inhibition observed in both quiescent stem cells and quiescent cancer cells appears to be a molecular event indispensable for cell dormancy. The targeted inhibition of mTOR has been recently demonstrated to be a mechanistic basis for cell hibernation in ESCs and cancer cells. The mTOR ATPase activity inhibitor INK128 that suppresses the catalytic activity of the mTOR subunit was found to effectively induce diapause in ESCs while maintaining full ESC viability in vitro and in vivo [9, 10]. Similarly, Rehman and colleagues induced a reversible state of dormancy in cancer cells, from which tumor cells emerged 14 days after withdrawal of the mTOR inhibitor INK128 [1].

Despite the findings highlighting the mTOR inhibition as a central molecular node in the transition of cancer cells to the latent phenotype, *modus operandi* of mTOR in the cancer cell dormancy is still regarded as both causal and paradoxical. Chemotherapy is well known to promote the emergence of dormant cancer cells, often referred to as drug-tolerant persisters [1, 15, 40] which are characterized by both down- [1, 13] and upregulated [41–43] mTOR signaling. The detection of both active and inactive mTOR causes confusion in understanding the legitimacy of mTOR suppression in dormant tumor cells. However, the data available in the literature provide insight into the proper interpretation of the role of mTOR signaling in the cancer cell dormancy. The cellular dormancy can be tentatively presented as temporary cell cycle arrest, quiescence, and senescence, with the mTOR pathway activated differently in these three types of cell quiescence. The mTORC1 activity has been shown to be required for the transition of stem cells from a quiescent state to a non-cyclic state primed to resume proliferation [7, 8, 11, 17]. Persistent mTOR signaling was observed in senescent somatic cells [44–46], with mTOR signaling suppressed in reversible hibernating cells. Cancer cell dormancy has not been clearly characterized biologically, with this term being referred to all types of nonproliferating drug-tolerant tumor cells. It is becoming evident that drug-tolerant persisters can co-opt quiescent signaling pathways in normal cells and can be somewhat distinguished based on the mTOR pathway activity. Dormant cancer cells with inhibited mTOR activity appears to be in a state of stable reversible hibernation, while active mTOR subunit indicates a temporary cell cycle arrest or senescence. Tumor persisters can display a senescence phenotype which is often correlated with an inhibited mTOR pathway [15, 47], again causing confusion in understanding the functions of mTOR in cancer cell dormancy. However, it was clearly demonstrated that the senescent phenotype did not prevent the exit of cancer cells from dormancy if their mTOR signaling was downregulated [15]. The authors reported that the pharmacological inhibition of mTOR induced the emergence of chemoresistant noncycling tumor cells that exhibited a senescence phenotype. The senescent cells were observed to re-establish the tumor population after removing the mTOR inhibitors, indicating that suppressed mTOR activity is as a strong determinant of recurrent cancer cells despite the evidence of senescence [15]. Therefore, a reversible chemoresistance phenotype can be stated to be regulated by the mTOR pathway, and the inhibition of mTOR signaling can be responsible for the tumor phenotype of the minimal residual disease following chemotherapy.

## Unresolved endoplasmic reticulum (ER) stress defines cell predisposition to dormancy through the prism of mTOR suppression

Cancer cell dissemination from the primary tumor and subsequent cancer cell survival are events regarded as catastrophic for patients because DTCs are the sources of metastatic cells, seeding various organs and tissues and often remaining latent there. The existing evidence indicates that metastasis precursors are not likely to originate from a specific preexisting clone in the primary tumor, as previously believed. They are more likely to result from phenotypic adaptation of tumor cells primed to enter a quiescent state [1, 13, 48, 49]. The available data suggest that this phenotypic adaptation involves intracellular mechanisms activated in normal cells during the detachment from the substrate.

The epithelial cells suspended for 24 h experience strong ER stress, triggering a massive activation of autophagy [50, 51]. ER stress is an ER environment disruption caused by numerous physiological and pathological factors, such as a substrate or nutrient deficiency or hypoxia, resulting in misfolded and unfolded protein accumulation in the ER [52]. This accumulation is followed by the so-called unfolded protein response (UPR) to restore cellular homeostasis or to induce apoptosis [53]. Three UPR pathways, namely, the PERK, IRE-1, and ATF6 pathways determine the subsequent cell fate under the ER stress [53]. ER-stressed epithelial cells were demonstrated to survive via canonical PERK activation [50, 51]. This kinase concurrently promotes autophagy and suppresses global protein synthesis via the eIF2α phosphorylation and mTOR inhibition [50, 51, 54]. Hence, PERK-dependent signaling is one of the major cytoprotective pathways in normal cells experiencing ER stress for some reasons.

The adaptation of DTCs during dissociation resembles the physiological strategy of normal cells to survive substrate loss by activating PERK signaling in response to ER stress. This resemblance was evidenced by the finding that DTCs in the bone marrow of cancer patients [55] and DTCs in mice models [3, 56] are characterized by sustained unresolved ER stress. The clinical detection of p38MAPK in latent cancer cells [19, 57] indicates the existence of chronic intracellular stress that is possibly the main trigger of dormancy-related pathways. Thus, the PERK-eIF2α signal transduction may represent a nodal point in molecular events from where the key pathways leading to quiescence start. The unresolved ER stress that does not induce cell death was demonstrated to ensure the physiological competence of DTCs to enter the dormant state in vitro and in vivo [3, 56]. Ranganathan and colleagues showed that the prolonged passaging of highly tumorigenic HEp3 cells in vitro resulted in the emergence of nonclonal cells that acquired a dormant phenotype upon inoculation in vivo [3]. High activation of the basal PERK-eIF2α pathway was indicated as the reason for the development of dormancy predisposition in vivo, and eIF2α phosphorylation mediated by PERK was found to be crucial for DTC survival and dormancy establishment in cancer cells [3].

Why is the PERK-eIF2α pathway so critical in shaping the cancer cell dormancy competence? The mTORC1 complex is localized at different subcellular compartments, such as lysosomes [58], ER [59], and Golgi [59] and links different metabolic pathways with external signals. Upon cell cycle arrest, mTORC1 is downregulated, stopping the biosynthetic processes through the inhibition of the S6K1 and 4E-BP1/eIF4E pathways. Incomplete protein synthesis is one of the triggers of the ER stress induction in cells, with different mTOR inhibitors being able to activate the PERK signaling in tumor cells [60]. If cells cannot restore proliferation, the unresolved ER stress further limits the mTORC1 activity [50, 61, 62], and the eIF2α phosphorylation by PERK provides the transition to alternative translation. The cap-independent mRNA translation is alternative mechanism of translation initiation, occurring at non-canonical start codons. This mechanism ensures that the translation of specific mRNAs occurs independently of mTOR [16, 63]. The eIF2α-dependent translation of mRNAs was demonstrated to be a fundamental mechanism of protein synthesis in quiescent stem cells [64]. The key events for the transition to alternative translation and autophagy activation occur in ER. These events are based on mTOR inhibition, underlying the decision to induce cell death or survive, i.e., to enter the dormant state under stress [62]. The experiments aimed at combination of the mTOR inhibition and ER stress induction followed by the mathematical modeling of cell effects observed revealed the interconnections between UPR and mTOR, which defined the dynamics of autophagy and apoptosis activation in cancer cells [62]. The authors showed that, under the ER stress, the active mTOR played a pro-apoptotic role, while the mTOR inhibition promoted cell viability by increasing the autophagic influx. The UPR target gene ATF4 is translated in a phospho-eIF2α-dependent manner and transcriptionally induces massive autophagy, ensuring cell viability under the ER stress [50, 63, 65, 66]. Active mTOR signaling can delay the transition to alternative translation of ATF4 mRNA and activation of related pathways, also impeding the selective translation of different genes required for a stress response in non-proliferating cells. Therefore, the capability of cancer cells to successfully activate massive autophagy, thereby ensuring viability, is determined by an effective transition to alternative translation underlying the transformation of tumor cells to a latent phenotype. This conclusion is also supported by the fact that incomplete mTOR pathway inhibition by rapamycin reduces the survival of blastocysts in the diapause state [9].

## Transition to alternative translation based on mTOR suppression provides new phenotypical traits in quiescent cancer cells

There is a substantial amount of evidence indicating that dormant/persisting cancer cells frequently exhibit molecular features similar to those of embryonic and adult stem cells. Hence, it has been postulated that recurrence is caused by the reactivation of dormant cancer stem cells [67, 68], which is supported by clinical findings demonstrating the detection of Nanog mRNA expression in dormant DTCs [19]. However, the results may also indicate that dormancy correlates with stem cell program activation in cancer cells.

**Fig. 2:**
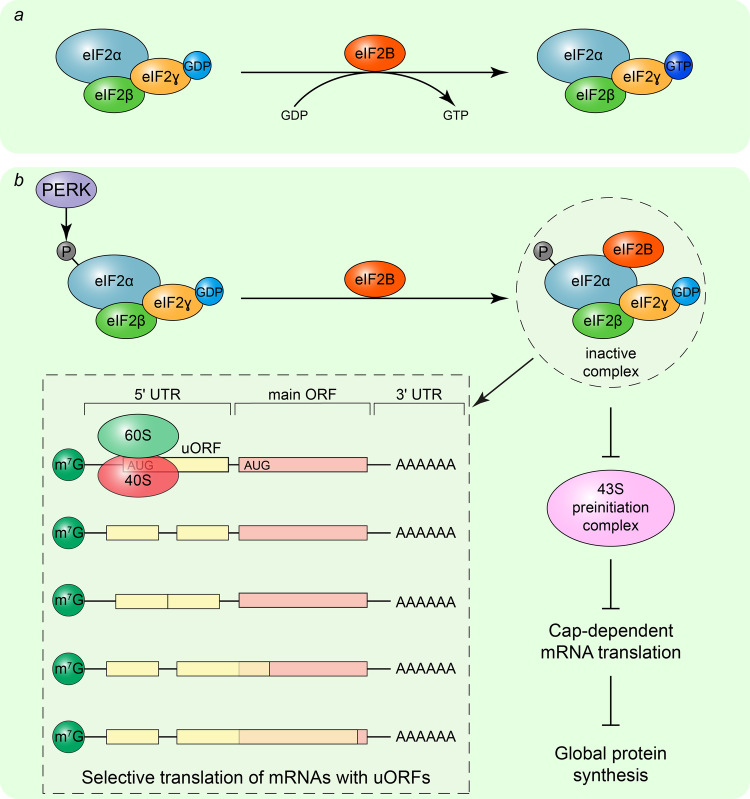
Scheme of selective translation of mRNAs with uORFs. **a** eIF2 exists in two distinct configurations: the GDP-bound (inactive) and GTP-bound (active) forms. The inactive-to-active conversation of eIF2 is catalyzed by the guanine nucleotide exchange factor eIF2B. In the absence of stress, eIF2 predominantly exists in the GTP-bound configuration and initiates cap-dependent translation. **b** In response to stress, PERK phosphorylates the α subunit of eIF2, which locks eIF2 into an inactive complex with eIF2B. As a result, the availability of the ternary complex for the formation of the 43S preinitiation complex is reduced, ultimately resulting in the global inhibition of translation. The translation is switched to the selective translation of mRNAs with uORFs to adapt to stress conditions. The translation of mRNA with uORFs is alternative mechanism of initiation of translation, and the 40S ribosome is recruited to the uORF regions which can contain the AUG start codon. The structure of uORFs can vary specifically in different genes, creating a unique translation pattern for each mRNA. uORFs-containing mRNA regions may overlap with the main reading frame.

The metabolic profiles of ESCs and adult stem cells are well-known to be strongly biased in the catabolic direction, with stem cells are characterized by low translational activity [69–72]. Although the stem cell genome is actively transcribed, mRNA levels correlate poorly with protein levels due to low translation efficiency [69]. Accordingly, mRNAs are translated in differentiated cells more effectively than in stem cells which produce less protein than their immediate descendants, as was showed in vivo [70, 71]. Several studies have strongly revealed that a low level of translation is critical for maintaining undifferentiated properties [69, 70], and the suppression of translational rate in differentiated cells triggers the stem phenotype automatically [72]. One of the intracellular mechanisms regulating a low protein synthesis is translation of mRNAs containing upstream open reading frames (uORFs) [73]. The ribosome behavior and the protein synthesis rate are primarily determined by the structure of the 5’ untranslated region in mRNA critical for ribosome recruitment and start codon selection (Fig. 2). uORFs often comprise start codon recognized by ribosome and tend to delay the ribosome progression to the main open reading frame and subsequent polypeptide synthesis [73, 74]. uORFs can be found in approximately half of human and mouse mRNA transcripts [73]. In ESCs and adult stem cells, the majority of translation initiation sites are located in uORFs, and such regulation significantly decreases during differentiation [75, 76]. One of the established mechanisms of the induction of selective mRNA translation via uORFs is eIF2α phosphorylation, with eIF2α-regulated protein translation being aprevalent pathway of protein biosynthesis in murine and human ESCs and adult stem cells [75]. Differentiated ESCs rarely use this mechanism to achieve proteostasis. The pharmacological inhibition of eIF2α phosphorylation in differentiated ESCs prevents differentiation by triggering stemness gene expression. Key pluripotency transcripts, such as dicer, lin28, trim71, CTCF, and Nanog, were demonstrated to be translated via uORFs in ESCs [77]. As such, ESCs enter the reversible dormant state without loss of stemness under mTOR inhibition because Oct4, Sox2, Nanog, Klf4, and other transcripts avoid mTOR-mediated translation repression. The protein synthesis in stem cells is primarily mTOR-independent. The mTOR kinase target 4E-BP1 was hyperphosphorylated in undifferentiated ESCs, indicating the downregulation of cap-dependent translation [70]. Complete inhibition of mTOR in ESCs can lead to enhanced pluripotency in human ESCs, approaching the one in human blastocysts [78].

**Fig. 3:**
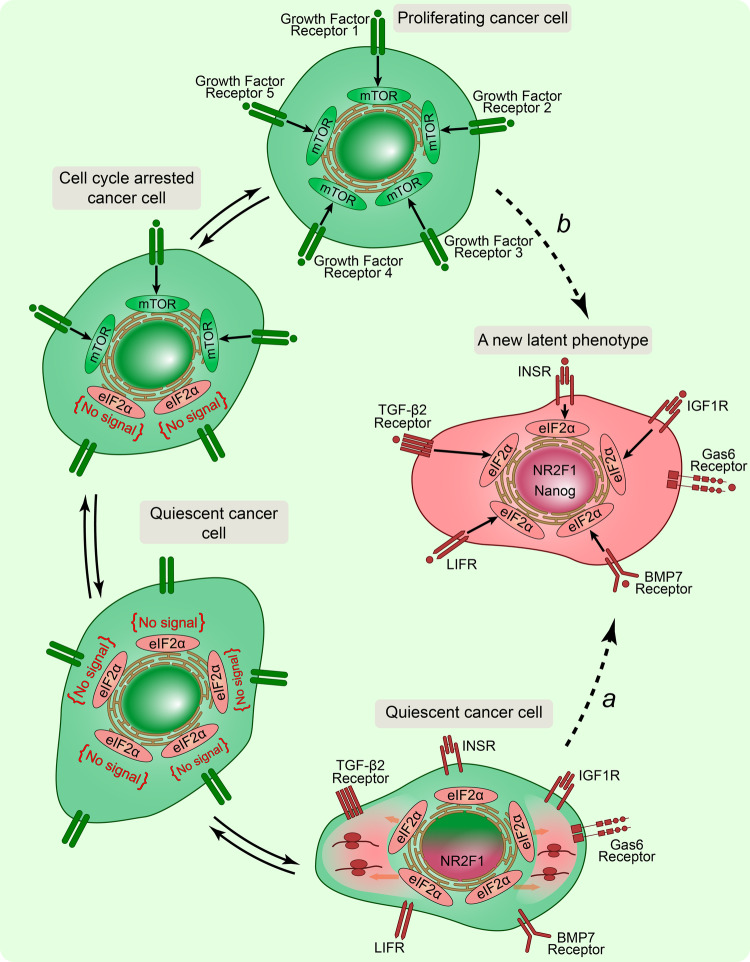
The dormancy concept dilemma: latent cancer cell versus quiescent cancer cell. **a** Translational reprogramming is considered as a non-genetic mechanism determining the emergence of a latent phenotype in cancer cells through dormancy transitions: via a cell cycle arrested state and a subsequent quiescent state. Proliferating cancer cell is highly sensitive to growth factor signals perceived by different types of growth factor receptors on cell surface, accordingly. Growth factors are critical extracellular stimuli regulating cell growth, proliferation, and differentiation, and, respectively, growth factor receptors were depicted as an example for illustration. Under starvation or various stress stimuli leading to decreasing signal transduction from growth factors, cancer cell undergoes cell cycle arrest accompanied by the mTOR activity restriction with switching to the eIF2α activation. Prolonged cell cycle arrest transits to a quiescent state based on the total reduction of mTOR activity and the induction of selective translational pathways. A quiescent cancer cell deprived of growth factor signals reprograms proteostasis in accordance with new environmental conditions expressing receptors on its surface serving for adaptation. Receptors such as BMP7 receptors, Gas6 receptors, TGF-β2 receptor, leukemia inhibitory factor receptor (LIFR) insulin receptor (INSR), and insulin-like growth factor 1 receptor (IGF1R) associated with cancer cell dormancy were taken as examples for illustration. Translational reprogramming can affect the gene regulatory network (the GRN) in a quiescent cancer cell and alter the epigenetic landscape, eventually leading to a new latent phenotype. Clinical cancer cell dormancy marker NR2F1 and Nanog can arise from the GRN shifting. Dormancy-associated receptors perceive specific stimuli and signal to the eIF2α pathway, critical for proteostasis in a latent cancer cell. **b** A latent phenotype in a cancer cell is the result of a direct irreversible transition from a proliferative phenotype based on epigenetic changes enabling translational reprogramming upon stress conditions. In this context, translational reprogramming is considered as a secondary process after epigenetic changes.

The available data suggest that mTOR inhibition and subsequent eIF2α phosphorylation as the key molecular axis ensuring stem cell proteostasis can promote stem-cell-like phenotypes in dormant cancer cells. mTOR inhibitor INK128 has been demonstrated to induce a reversible dormant state in colorectal cancer cells transcriptome of which is becoming similar to diapaused ESCs [1]. INK128 also induced the translation of NANOG, SNAIL, and NODAL mRNAs in breast cancer cells, enabling for the acquisition a phenotype towards to stemness [79]. Translation of proteins responsible for stem-like traits in breast cancer cells treated by INK128 was abrogated by drugs antagonizing eIF2α phosphorylation [79]. Accordingly, the activation of PERK-eIF2α signaling, a component of the ER stress response, can be a root of stemness in dormant cancer cells becoming capable of translating a subset of oncogenic proteins. However, given the generalized role of mTOR in metabolic processes and the number of identified complexes from 1 to 4, mTOR inhibition as a paramount molecular event can trigger distinct pathways of alternative translation besides the eIF2α pathway. eIF3D has been recently revealed as a critical upstream regulator of eIF2α phosphorylation in cells [80]. mTOR suppression induces eIF3D-mediated selective mRNA translation in non-proliferative tumor cells [16]. eIF3D activation ensured a latent phenotype in cancer cells with migratory features via eIF3D-dependent synthesis of proteins involved in cell motility [16]. As a result, cancer cells became noncycling and highly invasive. The eIF3D-dependent translation also enabled the synthesis of insulin receptors (INSR) and insulin-like growth factor 1 receptors (IGF1R), and the synthesis of proteins involved in the JAK/STAT pathway in non-dividing cancer cells [16]. The detection of active JAK/STAT signaling in these latent cells indicates that these cells highly respond to external stimuli. JAK/STAT signaling is an evolutionally conserved pathway for sensing diverse cytokines, interferons, growth factors, and related molecules [81]. It was demonstrated that the leukemia inhibitory factor (LIF) belonging to the IL-6 family of cytokines provided a pro-dormancy signal to tumor cells, with dormant cancer cells being LIF-responsive, compared to colonizing metastatic cells that did not respond to LIF [82–84]. From these investigations it follows that selective pathways of alternative translation can cause the acquisition of a new phenotype characterized by a certain set of receptors on the cell surface, stem-cell-like features, and motility. Hence, the widely used clinical term “latent metastatic cells” is the most appropriate to describe tumor cells of the minimal residual disease compared to quiescent/dormant cancer cells. The term “quiescent cancer cells” implies some inertness and weak cell sensitivity to external stimuli, including preservation of original properties, while latent cancer cells can be motile and sensitive to specific signals via activation of various selective translation pathways, resulting in the expression of factor-receptor proteins that perceive dormant stimuli. Thus, the concept of a quiescent state should be reconsidered in cancer cells and in stem cells, and it should be done in the context of translational reprogramming (Fig. 3).

A recent study has clearly demonstrated that non-genetic mechanisms associated with metabolic shifting can be a *prima causa* in establishing of the reversible drug-tolerant persister state in cancer cells [85]. Given that the absence of mTOR activity is characteristic of quiescent cells, and inhibition of mTOR kinase can alter the cellular proteome by 80% in cancer cells [16], translational reprogramming can be that one non-genetic mechanism determining the emergence of a latent phenotype in cancer cells and causing stem cell traits in dormant tumor cells. The inhibition of mTOR can rejuvenate cancer cells to some extent, like stem cells in which mTOR suppression enhances the differentiation potential [78, 86], and persister cancer cells generated during chemotherapy due to mTOR suppression can be regarded as a potential source of cancer stem cells upon resumption of proliferation. Compared to rejuvenated tumor cells, establishing a latent metastatic phenotype requires significant rebuilding the gene regulatory network (GRN), i.e., changes of stable patterns of gene expression that mutually regulate each other, keeping the cell in balance with the environment. It can be speculated that long-term mTOR blockade leads to the accumulation or exhaustion of specific proteins, which becomes limiting factor for the perturbation of local subnetworks in the GRN [87], resulting in new gene expression signatures described in the literature for dormant cancer cells, such as NR2F1, Nanog, and ZFP281 [14, 19, 23] (Fig. 3). Obviously, the mTOR inhibition per se is not sufficient for the GRN rearrangement, it is likely that the etiology of latent cancer cells is based on the synergy of external stimuli and mTOR blockage.

## Activation of autophagy and lysosomal biogenesis are long-range consequences of the molecular events of mTOR inhibition and PERK activation

Somatic cells and adult stem cells were demonstrated to gradually enter a quiescent state under stable dormant conditions, accompanied by gradual inhibition of transcriptional modules related to cell cycle progression, DNA replication, and other proliferation-associated pathways [25, 29, 30, 88, 89]. Accordingly, temporarily arrested cells are more quickly to resume proliferation than quiescent cells. RNA-sequencing analysis of 0- to 16-d serum-starved rat embryonic fibroblasts demonstrated a significant difference between slow-cycling and quiescent cells transcriptomes [88, 89]. The threshold of transition cells into quiescence was found to be determined by the levels of intensifications of autophagic and lysosomal fluxes [88, 89]. Autophagy and autophagic gene expression were continuously increased when the dormancy state in somatic and stem cells was deepened, with the lysosomal biogenesis is significantly induced, and lysosomal function is strengthened [25, 29, 30]. The disruptions in autophagy flow and/or lysosome functions in quiescent fibroblasts and stem cells caused the cells to enter an irreversible dormant state or senescence, which was defined as a state deeper than the quiescence one [25, 31, 89, 90]. This finding is well illustrated by an experiment with human fibroblasts that were exposed to contact inhibition for 150 days entered the long-term quiescence, culminating in senescence [91].

High autophagic activity is currently used as a clinical marker of dormant cancer cells and a general marker of quiescent stem cells, indicating that autophagy is a fundamental strategy for the survival of normal and malignant cells under the conditions of cellular quiescence [1, 25, 90, 92]. Unresolved ER stress is identified as the primary trigger of autophagy in quiescent cancer cells [50, 62]. However, the available data also suggest that in quiescent stem and cancer cells, autophagy is massively induced by the transcription factor TFEB required for cell survival during prolonged cell cycle blockade [25, 30, 93]. TFEB has been recognized as a key regulator of the reproductive quiescence of *C. elegans*: it induces major transcriptomic changes in cells, enabling morphological and physiological remodeling of organisms required to enter the quiescent state and survive during the one [94]. TFEB is accumulated in the nuclei of deeply hibernating stem cells, activating autophagic and lysosomal gene transcription by inducing constitutive autophagic-lysosomal flow [25, 30, 31]. TFEB activation in quiescent stem cells leads to strong stimulation of catabolic pathways and enhances degradation of signaling receptors and nutrient receptors, restricting mitogenic activation of dormant cells and defining long-term hibernation regime [25, 30, 31]. Thus, TFEB has been defined as the part of the mechanism by which stem cells can remain quiescent for months and years in adult organisms.

The data obtained on the involvement of TFEB in cellular dormancy are few and do not provide a complete understanding of all its functions. A basic mechanism of TFEB regularity in cells has been defined as: TFEB is localized at lysosomes in the absence of stress, where mTORC1 negatively regulates TFEB via the Rag GTPases, preventing its nuclear translocation [95, 96]. Thus, the suppression of mTOR in quiescent cells can be assumed to a priori cause the activation of TFEB and its transcriptional functions. This assumption is supported by the fact that pharmacological inhibition of the mTOR kinase subunit in cells activates TFEB to be translocated into the nucleus to induce the expression of target genes [95]. In addition, ER stress also stimulates TFEB activation in cells. However, nuclear translocation of TFEB under ER stress can be mTORC1-independent but may require PERK [97]. Should one attempt to reconstruct the molecular events leading to cellular quiescence, the molecular strategies will overlap in the following way: the TFEB pathway becomes activated in response to prolonged ER stress and mTOR inhibition but requires the MYC transcription module to be suppressed to enhance autophagy and lysosomal biogenesis. This reconstruction of molecular events provides insight into where to search for clinical markers of dormant cancer cells responsible for minimal residual disease. Dormancy-specific lysosomal enzymes are most likely to be detected after the TFEB-dependent lysosome biogenesis. The point is that lysosomal enzymes in cells under dormancy are synthesized under mTOR inhibition and are translated by selective translation. Accordingly, the composition of lysosomal enzymes may significantly differ in proliferating cells or temporarily arrested cells compared to quiescent cells characterized by an abundance of large lysosomes. Therefore, further investigations of targeted mTOR inhibition will allow one to study the transition of tumor cells to selective translation of mRNAs and explore the activation of TFEB-dependent autophagy and lysosomes with a focus on lysosomes.

## Conclusion

**Fig. 4:**
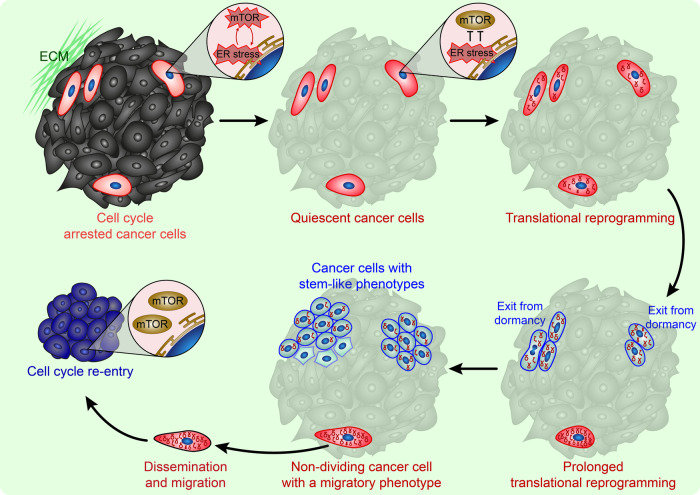
Clinical scenario of cancer cell development through the mTOR inhibition-based dormant transitions. During chemotherapy, ER stress occurs in cell cycle-arrested tumor cells, leading to suppression of the mTOR pathway in some cancer cells. The sustained suppression of the mTOR pathway leads to a quiescent state. Quiescent cancer cells undergo translational reprogramming and accumulate oncogenic proteins, resulting in populations of cancer stem cells after resumption of proliferation. Long-term quiescent cancer cells can undergo major transformation based on translational reprogramming to form a latent metastatic phenotype. Cancer stem cells may repeat dormant transitions to form new latent metastatic cells. A latent metastatic cells exhibit the suppressed mTOR signaling which restore in the appropriate conditions with producing next generation of cancer cells.

One of the cornerstones of modern oncology is understanding the mechanisms underlying the development of metastatic cells and their dependence on a latent state. Would-be metastatic cells were previously thought to originate from DTCs with specific mutations, with tumor cell evolution being interpreted by mutational changes. Later, it was established that DTCs emerged due to phenotypic adaptation, making it evident that the evolution of tumor cells also occurs via the selection of their opportunistic properties. The clinical scenario of the latent metastatic cell progression may become clear if one applies the accumulated knowledge about the mTOR signaling pattern in dormant cancer cells (Fig. 4). The arguments provided in this review strongly support the idea that inhibition of mTOR kinase in tumor cells during chemotherapy may be a major evolutionary vector for cancer cell phenotypic adaptation within the body. The mTOR kinase suppression results in a switch to alternative translation of proteins, ensuring plasticity and mobility in tumor cells incapable of proliferation. The duration of mTOR suppression-based quiescence may determine the phenotypic outcomes in tumor cells after resumption to proliferation. To put it another way, the prolonged blockade of the mTOR pathway leads to a chronic intracellular stress response associated with enhancing the alternative biosynthesis pathways and the accumulation of proteins counteracting stress. The composition and functions of accumulated proteins may vary depending on the selective translation types, their regulation, and intensification. Hence, non-proliferating tumor cells develop high resistance to environmental factors, increasing their oncogenic potential during the dormancy period. As a result, cancer cells with stem-like characteristics resume proliferation after the recovery of mTOR activity, explaining the phenotypical diversity of reversible drug-tolerant cells.

Such licensed drugs for use in a variety of cancers as rapamycin and its derivatives sirolimus, temsirolimus, and everolimus are allosteric inhibitors of mTORC1. But allosteric suppression of mTORC1 often causes a feed-back loops of prosurvival ERK1/2 [98] and AKT [99] signaling in cancer cells obtained from patients. Hence, preclinical and clinical data support the use of mTOR inhibitors with broader activity (TORC1 and TORC2), and such dual mTORC1/2 selective ATP competitive inhibitor as AZD2014 (Vistusertib) is clinical candidate with optimal pharmacokinetics and pharmacodynamics compared to other dual mTORC1/2 inhibitors [100–103]. To date, some clinical trials of AZD2014 have completed phase I evaluation and have moved to phase II evaluation [102]. Evidence that mTOR suppression leads to a dormant state in tumor cells followed by translational reprogramming should be taken into account under cancer treatment with dual mTORC1/2 inhibitors to prevent possible fatal consequences in patients.

## References

[1] Rehman SK, Haynes J, Collignon E, Brown KR, Wang Y, Nixon AML (2021). Colorectal cancer cells enter a diapause-like DTP state to survive chemotherapy. Cell.

[2] Aguirre-Ghiso JA, Liu D, Mignatti A, Kovalski K, Ossowski L (2001). Urokinase receptor and fibronectin regulate the ERKMAPK to p38MAPK activity ratios that determine carcinoma cell proliferation or dormancy in vivo. Mol Biol Cell.

[3] Ranganathan AC, Ojha S, Kourtidis A, Conklin DS, Aguirre-Ghiso JA (2008). Dual function of pancreatic endoplasmic reticulum kinase in tumor cell growth arrest and survival. Cancer Res.

[4] Li L, Clevers H (2010). Coexistence of quiescent and active adult stem cells in mammals. Science.

[5] Chen C, Liu Y, Liu R, Ikenoue T, Guan K-L, Liu Y (2008). TSC-mTOR maintains quiescence and function of hematopoietic stem cells by repressing mitochondrial biogenesis and reactive oxygen species. J Exp Med.

[6] Adhikari D, Zheng W, Shen Y, Gorre N, Hämäläinen T, Cooney AJ (2010). Tsc/mTORC1 signaling in oocytes governs the quiescence and activation of primordial follicles. Hum Mol Genet.

[7] Rodgers JT, King KY, Brett JO, Cromie MJ, Charville GW, Maguire KK (2014). mTORC1 controls the adaptive transition of quiescent stem cells from G0 to GAlert. Nature.

[8] Deng Z, Lei X, Zhang X, Zhang H, Liu S, Chen Q (2015). mTOR signaling promotes stem cell activation via counterbalancing BMP-mediated suppression during hair regeneration. J Mol Cell Biol.

[9] Bulut-Karslioglu A, Biechele S, Jin H, Macrae TA, Hejna M, Gertsenstein M (2016). Inhibition of mTOR induces a paused pluripotent state. Nature.

[10] Hussein AM, Wang Y, Mathieu J, Margaretha L, Song C, Jones DC (2020). Metabolic control over mTOR-dependent diapause-like state. Dev Cell.

[11] Nieto-González JL, Gómez-Sánchez L, Mavillard F, Linares-Clemente P, Rivero MC, Valenzuela-Villatoro M (2019). Loss of postnatal quiescence of neural stem cells through mTOR activation upon genetic removal of cysteine string protein-α. Proc Natl Acad Sci USA.

[12] Kim JK, Jung Y, Wang J, Joseph J, Mishra A, Hill EE (2013). TBK1 regulates prostate cancer dormancy through mTOR inhibition. Neoplasia.

[13] Dhimolea E, de Matos Simoes R, Kansara D, Al’Khafaji A, Bouyssou J, Weng X (2021). An embryonic diapause-like adaptation with suppressed Myc activity enables tumor treatment persistence. Cancer Cell.

[14] Khalil BD, Sanchez R, Rahman T, Rodriguez-Tirado C, Moritsch S, Martinez AR (2021). An NR2F1-specific agonist suppresses metastasis by inducing cancer cell dormancy. J Exp Med.

[15] Liu Y, Azizian NG, Sullivan DK, Li Y (2022). mTOR inhibition attenuates chemosensitivity through the induction of chemotherapy resistant persisters. Nat Commun.

[16] Shin S, Han M-J, Jedrychowski MP, Zhang Z, Shokat KM, Plas DR (2023). mTOR inhibition reprograms cellular proteostasis by regulating eIF3D-mediated selective mRNA translation and promotes cell phenotype switching. Cell Rep.

[17] Kellenberger AJ, Tauchi M (2013). Mammalian target of rapamycin complex 1 (mTORC1) may modulate the timing of anagen entry in mouse hair follicles. Exp Dermatol.

[18] Rodriguez-Tirado C, Kale N, Carlini MJ, Shrivastava N, Rodrigues AA, Khalil B (2022). NR2F1 is a barrier to dissemination of early stage breast cancer cells. Cancer Res.

[19] Sosa MS, Parikh F, Maia AG, Estrada Y, Bosch A, Bragado P (2015). NR2F1 controls tumour cell dormancy via SOX9- and RARβ-driven quiescence programmes. Nat Commun.

[20] Gao X, Zhang M, Tang Y, Liang X (2017). Cancer cell dormancy: mechanisms and implications of cancer recurrence and metastasis. Onco Targets Ther.

[21] Borgen E, Rypdal MC, Sosa MS, Renolen A, Schlichting E, Lønning PE (2018). NR2F1 stratifies dormant disseminated tumor cells in breast cancer patients. Breast Cancer Res.

[22] Cole AJ, Iyengar M, Panesso-Gómez S, O'Hayer P, Chan D, Delgoffe GM (2020). NFATC4 promotes quiescence and chemotherapy resistance in ovarian cancer. JCI Insight.

[23] Nobre AR, Dalla E, Yang J, Huang X, Wullkopf L, Risson E (2022). ZFP281 drives a mesenchymal-like dormancy program in early disseminated breast cancer cells that prevents metastatic outgrowth in the lung. Nat Cancer.

[24] Scognamiglio R, Cabezas-Wallscheid N, Thier MC, Altamura S, Reyes A, Prendergast ÁM (2016). Myc depletion induces a pluripotent dormant state mimicking diapause. Cell.

[25] García-Prat L, Kaufmann KB, Schneiter F, Voisin V, Murison A, Chen J (2021). TFEB-mediated endolysosomal activity controls human hematopoietic stem cell fate. Cell Stem Cell.

[26] Marqués-Torrejón MÁ, Williams CAC, Southgate B, Alfazema N, Clements MP, Garcia-Diaz C (2021). LRIG1 is a gatekeeper to exit from quiescence in adult neural stem cells. Nat Commun.

[27] Cai C, Hu X, Dai P, Zhang T, Jiang M, Wang L (2021). c-Myc regulates neural stem cell quiescence and activation by coordinating the cell cycle and mitochondrial remodeling. Sig Transduct Target Ther.

[28] Wilson A, Laurenti E, Oser G, van der Wath RC, Blanco-Bose W, Jaworski M (2008). Hematopoietic stem cells reversibly switch from dormancy to self-renewal during homeostasis and repair. Cell.

[29] Liang R, Arif T, Kalmykova S, Kasianov A, Lin M, Menon V (2020). Restraining lysosomal activity preserves hematopoietic stem cell quiescence and potency. Cell Stem Cell.

[30] Kobayashi T, Piao W, Takamura T, Kori H, Miyachi H, Kitano S (2019). Enhanced lysosomal degradation maintains the quiescent state of neural stem cells. Nat Commun.

[31] Leeman DS, Hebestreit K, Ruetz T, Webb AE, McKay A, Pollina EA (2018). Lysosome activation clears aggregates and enhances quiescent neural stem cell activation during aging. Science.

[32] Raben N, Puertollano R (2016). TFEB and TFE3: linking lysosomes to cellular adaptation to stress. Annu Rev Cell Dev Biol.

[33] Palmieri M, Pal R, Nelvagal HR, Lotfi P, Stinnett GR, Seymour ML (2017). mTORC1-independent TFEB activation via Akt inhibition promotes cellular clearance in neurodegenerative storage diseases. Nat Commun.

[34] La T, Chen S, Guo T, Zhao XH, Teng L, Li D (2021). Visualization of endogenous p27 and Ki67 reveals the importance of a c-Myc-driven metabolic switch in promoting survival of quiescent cancer cells. Theranostics.

[35] Harwood FC, Klein Geltink RI, O’Hara BP, Cardone M, Janke L, Finkelstein D (2018). ETV7 is an essential component of a rapamycin-insensitive mTOR complex in cancer. Sci Adv.

[36] Nguyen JT, Haidar FS, Fox AL, Ray C, Mendonça DB, Kim JK (2019). mEAK-7 forms an alternative mTOR complex with DNA-PKcs in human cancer. iScience.

[37] Raught B, Peiretti F, Gingras A-C, Livingstone M, Shahbazian D, Mayeur GL (2004). Phosphorylation of eucaryotic translation initiation factor 4B Ser422 is modulated by S6 kinases. EMBO J.

[38] Meyuhas O (2000). Synthesis of the translational apparatus is regulated at the translational level. Eur J Biochem.

[39] Ma XM, Blenis J (2009). Molecular mechanisms of mTOR-mediated translational control. Nat Rev Mol Cell Biol.

[40] Vo T-TT, Lee JS, Nguyen D, Lui B, Pandori W, Khaw A (2017). mTORC1 inhibition induces resistance to methotrexate and 6-mercaptopurine in Ph+ and Ph-like B-ALL. Mol Cancer Ther.

[41] Schewe DM, Aguirre-Ghiso JA (2008). ATF6α-Rheb-mTOR signaling promotes survival of dormant tumor cells in vivo. Proc Natl Acad Sci USA.

[42] Boral D, Liu HN, Kenney SR, Marchetti D (2020). Molecular Interplay between dormant bone marrow-resident cells (BMRCs) and CTCs in breast cancer. Cancers.

[43] Pallis M, Harvey T, Russell N (2016). Phenotypically dormant and immature leukaemia cells display increased ribosomal protein S6 phosphorylation. PLoS ONE.

[44] Carroll B, Nelson G, Rabanal-Ruiz Y, Kucheryavenko O, Dunhill-Turner NA, Chesterman CC (2017). Persistent mTORC1 signaling in cell senescence results from defects in amino acid and growth factor sensing. J Cell Biol.

[45] Park JH, Lee NK, Lim HJ, Ji Staek, Kim Y-J, Jang WB (2020). Pharmacological inhibition of mTOR attenuates replicative cell senescence and improves cellular function via regulating the STAT3-PIM1 axis in human cardiac progenitor cells. Exp Mol Med.

[46] Herranz N, Gallage S, Mellone M, Wuestefeld T, Klotz S, Hanley CJ (2015). mTOR regulates MAPKAPK2 translation to control the senescence-associated secretory phenotype. Nat Cell Biol.

[47] Jochems F, Thijssen B, Conti GD, Jansen R, Pogacar Z, Groot K (2021). The cancer SENESCopedia: a delineation of cancer cell senescence. Cell Rep..

[48] Schmidt-Kittler O, Ragg T, Daskalakis A, Granzow M, Ahr A, Blankenstein TJF (2003). From latent disseminated cells to overt metastasis: genetic analysis of systemic breast cancer progression. Proc Natl Acad Sci USA.

[49] Malladi S, Macalinao DG, Jin X, He L, Basnet H, Zou Y (2016). Metastatic latency and immune evasion through autocrine inhibition of WNT. Cell.

[50] Avivar-Valderas A, Salas E, Bobrovnikova-Marjon E, Diehl JA, Nagi C, Debnath J (2011). PERK integrates autophagy and oxidative stress responses to promote survival during extracellular matrix detachment. Mol Cell Biol.

[51] Avivar-Valderas A, Bobrovnikova-Marjon E, Diehl JA, Bardeesy N, Debnath J, Aguirre-Ghiso J (2013). Regulation of autophagy during ECM detachment is linked to a selective inhibition of mTORC1 by PERK. Oncogene.

[52] Tabas I, Ron D (2011). Integrating the mechanisms of apoptosis induced by endoplasmic reticulum stress. Nat Cell Biol.

[53] Ron D, Walter P (2007). Signal integration in the endoplasmic reticulum unfolded protein response. Nat Rev Mol Cell Biol.

[54] Sequeira SJ, Ranganathan AC, Adam AP, Iglesias BV, Farias EF, Aguirre-Ghiso JA (2007). Inhibition of proliferation by PERK regulates mammary acinar morphogenesis and tumor formation. PLoS ONE.

[55] Bartkowiak K, Kwiatkowski M, Buck F, Gorges TM, Nilse L, Assmann V (2015). Disseminated tumor cells persist in the bone marrow of breast cancer patients through sustained activation of the unfolded protein response. Cancer Res.

[56] Pommier A, Anaparthy N, Memos N, Kelley ZL, Gouronnec A, Yan R (2018). Unresolved endoplasmic reticulum stress engenders immune-resistant, latent pancreatic cancer metastases. Science.

[57] Chéry L, Lam H-M, Coleman I, Lakely B, Coleman R, Larson S (2014). Characterization of single disseminated prostate cancer cells reveals tumor cell heterogeneity and identifies dormancy associated pathways. Oncotarget.

[58] Settembre C, Di Malta C, Polito VA, Arencibia MG, Vetrini F, Erdin S (2011). TFEB links autophagy to lysosomal biogenesis. Science.

[59] Liu X, Zheng XFS (2007). Endoplasmic reticulum and Golgi localization sequences for mammalian target of rapamycin. Mol Biol Cell.

[60] Freis P, Bollard J, Lebeau J, Massoma P, Fauvre J, Vercherat C (2017). mTOR inhibitors activate PERK signaling and favor viability of gastrointestinal neuroendocrine cell lines. Oncotarget.

[61] Mafi S, Ahmadi E, Meehan E, Chiari C, Mansoori B, Sadeghi H (2023). The mTOR signaling pathway interacts with the ER stress response and the unfolded protein response in cancer. Cancer Res.

[62] Kapuy O, Vinod PK, Bánhegyi G (2014). mTOR inhibition increases cell viability via autophagy induction during endoplasmic reticulum stress - An experimental and modeling study. FEBS Open Bio.

[63] Vattem KM, Wek RC (2004). Reinitiation involving upstream ORFs regulates ATF4 mRNA translation in mammalian cells. Proc Natl Acad Sci USA.

[64] Zismanov V, Chichkov V, Colangelo V, Jamet S, Wang S, Syme A (2016). Phosphorylation of eIF2α Is a translational control mechanism regulating muscle stem cell quiescence and self-renewal. Cell Stem Cell.

[65] B’chir W, Maurin A-C, Carraro V, Averous J, Jousse C, Muranishi Y (2013). The eIF2α/ATF4 pathway is essential for stress-induced autophagy gene expression. Nucleic Acids Res.

[66] Rzymski T, Milani M, Pike L, Buffa F, Mellor HR, Winchester L (2010). Regulation of autophagy by ATF4 in response to severe hypoxia. Oncogene.

[67] Talukdar S, Bhoopathi P, Emdad L, Das S, Sarkar D, Fisher PB (2019). Dormancy and cancer stem cells: an enigma for cancer therapeutic targeting. Adv Cancer Res.

[68] Kleffel S, Schatton T (2013). Tumor dormancy and cancer stem cells: two sides of the same coin?. Adv Exp Med Biol.

[69] Signer RAJ, Magee JA, Salic A, Morrison SJ (2014). Haematopoietic stem cells require a highly regulated protein synthesis rate. Nature.

[70] Sampath P, Pritchard DK, Pabon L, Reinecke H, Schwartz SM, Morris DR (2008). A hierarchical network controls protein translation during murine embryonic stem cell self-renewal and differentiation. Cell Stem Cell.

[71] Blanco S, Bandiera R, Popis M, Hussain S, Lombard P, Aleksic J (2016). Stem cell function and stress response are controlled by protein synthesis. Nature.

[72] Blanco S, Kurowski A, Nichols J, Watt FM, Benitah SA, Frye M (2011). The RNA-methyltransferase Misu (NSun2) poises epidermal stem cells to differentiate. PLoS Genet.

[73] Calvo SE, Pagliarini DJ, Mootha VK (2009). Upstream open reading frames cause widespread reduction of protein expression and are polymorphic among humans. Proc Natl Acad Sci USA.

[74] Lin Y, May GE, Kready H, Nazzaro L, Mao M, Spealman P (2019). Impacts of uORF codon identity and position on translation regulation. Nucleic Acids Res.

[75] Friend K, Brooks HA, Propson NE, Thomson JA, Kimble J (2015). Embryonic stem cell growth factors regulate eIF2α phosphorylation. PLoS ONE.

[76] Ingolia NT, Lareau LF, Weissman JS (2011). Ribosome profiling of mouse embryonic stem cells reveals the complexity of mammalian proteomes. Cell.

[77] Popa A, Lebrigand K, Barbry P, Waldmann R (2016). Pateamine A-sensitive ribosome profiling reveals the scope of translation in mouse embryonic stem cells. BMC Genomics.

[78] Hu Z, Li H, Jiang H, Ren Y, Yu X, Qiu J (2020). Transient inhibition of mTOR in human pluripotent stem cells enables robust formation of mouse-human chimeric embryos. Sci Adv.

[79] Jewer M, Lee L, Leibovitch M, Zhang G, Liu J, Findlay SD (2020). Translational control of breast cancer plasticity. Nat Commun.

[80] Mukhopadhyay S, Amodeo ME, Lee ASY (2023). eIF3d controls the persistent integrated stress response. Mol Cell.

[81] Liongue C, O’Sullivan LA, Trengove MC, Ward AC (2012). Evolution of JAK-STAT pathway components: mechanisms and role in immune system development. PLoS ONE.

[82] Johnson RW, Finger EC, Olcina MM, Vilalta M, Aguilera T, Miao Y (2016). Induction of LIFR confers a dormancy phenotype in breast cancer cells disseminated to the bone marrow. Nat Cell Biol.

[83] Chen D, Sun Y, Wei Y, Zhang P, Rezaeian AH, Teruya-Feldstein J (2012). LIFR is a breast cancer metastasis suppressor upstream of the Hippo-YAP pathway and a prognostic marker. Nat Med.

[84] Iorns E, Ward TM, Dean S, Jegg A, Thomas D, Murugaesu N (2012). Whole genome in vivo RNAi screening identifies the leukemia inhibitory factor receptor as a novel breast tumor suppressor. Breast Cancer Res Treat.

[85] Oren Y, Tsabar M, Cuoco MS, Amir-Zilberstein L, Cabanos HF, Hütter J-C (2021). Cycling cancer persister cells arise from lineages with distinct programs. Nature.

[86] Hartman NW, Lin TV, Zhang L, Paquelet GE, Feliciano DM, Bordey A (2013). mTORC1 targets the translational repressor 4E-BP2, but not S6 kinase 1/2, to regulate neural stem cell self-renewal in vivo. Cell Rep..

[87] Uzuner D, Akkoç Y, Peker N, Pir P, Gözüaçık D, Çakır T (2021). Transcriptional landscape of cellular networks reveal interactions driving the dormancy mechanisms in cancer. Sci Rep..

[88] Kwon JS, Everetts NJ, Wang X, Wang W, Della Croce K, Xing J (2017). Controlling depth of cellular quiescence by an Rb-E2F network switch. Cell Rep..

[89] Fujimaki K, Li R, Chen H, Della Croce K, Zhang HH, Xing J (2019). Graded regulation of cellular quiescence depth between proliferation and senescence by a lysosomal dimmer switch. Proc Natl Acad Sci USA.

[90] Ho TT, Warr MR, Adelman ER, Lansinger OM, Flach J, Verovskaya EV (2017). Autophagy maintains the metabolism and function of young and old stem cells. Nature.

[91] Marthandan S, Priebe S, Hemmerich P, Klement K, Diekmann S (2014). Long-term quiescent fibroblast cells transit into senescence. PLoS ONE.

[92] Vera-Ramirez L, Vodnala SK, Nini R, Hunter KW, Green JE (2018). Autophagy promotes the survival of dormant breast cancer cells and metastatic tumour recurrence. Nat Commun.

[93] Zangrossi M, Romani P, Chakravarty P, Ratcliffe CDH, Hooper S, Dori M (2021). EphB6 regulates TFEB-lysosomal pathway and survival of disseminated indolent breast cancer cells. Cancers.

[94] Gerisch B, Tharyan RG, Mak J, Denzel SI, Popkes-van Oepen T, Henn N (2020). HLH-30/TFEB is a master regulator of reproductive quiescence. Dev Cell.

[95] Settembre C, Zoncu R, Medina DL, Vetrini F, Erdin S, Erdin S (2012). A lysosome-to-nucleus signalling mechanism senses and regulates the lysosome via mTOR and TFEB. EMBO J.

[96] Cui Z, Napolitano G, de Araujo MEG, Esposito A, Monfregola J, Huber LA (2023). Structure of the lysosomal mTORC1–TFEB–Rag–Ragulator megacomplex. Nature.

[97] Martina JA, Diab HI, Brady OA, Puertollano R (2016). TFEB and TFE3 are novel components of the integrated stress response. EMBO J.

[98] Carracedo A, Ma L, Teruya-Feldstein J, Rojo F, Salmena L, Alimonti A (2008). Inhibition of mTORC1 leads to MAPK pathway activation through a PI3K-dependent feedback loop in human cancer. J Clin Invest.

[99] O’Reilly KE, Rojo F, She Q-B, Solit D, Mills GB, Smith D (2006). mTOR inhibition induces upstream receptor tyrosine kinase signaling and activates Akt. Cancer Res.

[100] Pike KG, Malagu K, Hummersone MG, Menear KA, Duggan HME, Gomez S (2013). Optimization of potent and selective dual mTORC1 and mTORC2 inhibitors: the discovery of AZD8055 and AZD2014. Bioorg Med Chem Lett.

[101] Basu B, Krebs MG, Sundar R, Wilson RH, Spicer J, Jones R (2018). Vistusertib (dual m-TORC1/2 inhibitor) in combination with paclitaxel in patients with high-grade serous ovarian and squamous non-small-cell lung cancer. Ann Oncol.

[102] Eyre TA, Hildyard C, Hamblin A, Ali AS, Houlton A, Hopkins L (2019). A phase II study to assess the safety and efficacy of the dual mTORC1/2 inhibitor vistusertib in relapsed, refractory DLBCL. Hematol Oncol.

[103] Morscher RJ, Brard C, Berlanga P, Marshall LV, André N, Rubino J (2021). First-in-child phase I/II study of the dual mTORC1/2 inhibitor vistusertib (AZD2014) as monotherapy and in combination with topotecan-temozolomide in children with advanced malignancies: arms E and F of the AcSé-ESMART trial. Eur J Cancer.

